# The critical role of primary care clinicians in the early detection of ocular surface squamous neoplasia

**DOI:** 10.4102/safp.v67i1.6065

**Published:** 2025-02-10

**Authors:** Leendert Dekker, Jan F. Olivier, Klaus von Pressentin

**Affiliations:** 1 Department of Ophthalmology, Faculty of Medicine, Dr George Mukhari Academic Hospital, City of Tshwane, South Africa; 2 Department of Ophthalmology, Faculty of Medicine, Sefako Makgatho Health Sciences University, City of Tshwane, South Africa; 3 Division of Family Medicine, Department of Family, Community and Emergency Care, Faculty of Health Sciences, University of Cape Town, Cape Town, South Africa

**Keywords:** ocular surface squamous neoplasia, OSSN, HPV, HIV, ultraviolet radiation, primary care

## Abstract

Ocular surface squamous neoplasia (OSSN) encompasses a spectrum of conjunctival tumours and, while rare globally, is the most common ocular malignancy in sub-Saharan Africa. Its rising incidence, primarily driven by the human immunodeficiency virus (HIV) epidemic, presents significant challenges in clinical diagnosis, as these lesions often share characteristics with other conjunctival lesions. In South Africa, where risk factors such as HIV, human papillomavirus infection and excessive sun exposure are prevalent, primary care clinicians play a crucial role in identifying and referring cases for early intervention. Ocular surface squamous neoplasia is often the first indication of HIV in patients who are otherwise unaware of their status when they present to healthcare workers, making it essential for healthcare workers to screen for HIV and initiate antiretroviral therapy. Early recognition of at-risk patients and prompt referral of suspicious lesions are imperative to improve patient outcomes and prevent vision loss.

## Introduction

Ocular surface squamous neoplasia (OSSN) is a broad term that includes all squamous carcinomas of the conjunctiva, encompassing conjunctival intraepithelial lesions, carcinoma *in situ* and invasive squamous cell carcinoma.^1,2,3,4^ While OSSN has a low mortality rate and is generally curable, delayed diagnosis or misdiagnosis can lead to significant complications, including vision loss, local invasion and metastasis, thereby imposing medical and economic burdens.^2,5^ Primary care clinicians are often the first point of contact for underserved and vulnerable communities, where access to specialist care may be limited. Their awareness of OSSN is essential to facilitate early referral, timely diagnosis and prevent severe complications.

## Epidemiology

Ocular surface squamous neoplasia is recognised in two distinct categories. Firstly, in temperate climates, it predominantly affects older Caucasian men in their seventh decade and tends to grow slowly. Secondly, in tropical climates, both men and women in their thirties are equally affected, with the condition often linked to human papillomavirus (HPV) and human immunodeficiency virus (HIV) infections.^1,6^

Incidence estimates of OSSN vary regionally.^6,7^ In the United States (US) and Australia, the incidence ranges from 0.03 to 1.9 cases per 100 000 persons per year.^1^ While OSSN remains rare globally, it is the most common ocular tumour in sub-Saharan Africa.^1,8^ The HIV pandemic has significantly contributed to its rising incidence in this region,^1,8^ with reported rates ranging from 1.6 to 3.4 cases per 100 000 persons annually.^1^ Notably, there is a reported 12-fold increase in OSSN among patients infected with HIV in the US,^9^ with the highest risk occurring within the first 2 years following an acquired immunodeficiency syndrome (AIDS) diagnosis.^2^

Patients presenting with OSSN can be unaware of their HIV status.^8,10^ In 50% – 86% of cases, OSSN has been reported as the first clinical sign of HIV and/or AIDS.^2,8,10,11^ In Uganda, 65% of HIV-infected patients with OSSN died from AIDS-related complications, with a median survival of 20 months following their OSSN diagnosis.^12^ The impact of antiretroviral therapy (ART) on OSSN remains uncertain.^10^ A study by Guech-Ongey, examining patients on highly active antiretroviral therapy (HAART), found no significant difference in the occurrence of OSSN between those receiving HAART and those not on treatment.^9^ The availability of ART and the extended survival of individuals with HIV may have contributed to the rising incidence of OSSN.^10^ Human immunodeficiency virus not only increases the risk of developing OSSN in a younger population but also heightens the likelihood of more extensive lesions, higher-grade malignancy, bilateral disease and a higher recurrence rate.^2,10^

## Pathophysiology

Ocular surface squamous neoplasia is characterised by dysplastic cells occupying the conjunctival epithelium from the basal layer upwards.^1,5^ When the epithelial basal layer remains intact, the lesion is classified as a conjunctival intraepithelial neoplasia (CIN) or carcinoma *in situ* if the entire conjunctival epithelium is affected.^5,11^ In cases where the basal layer is breached, the condition progresses to invasive squamous cell carcinoma.^1,5,11^

The aetiology of OSSN is thought to result from a complex interplay of genetic and environmental factors.^1,13^ Major risk factors include ultraviolet radiation (UVR) exposure, immunosuppression, the oncogenic potential of HPV infection and alterations in the p53 tumour suppressor gene.^1,2,13^

Ultraviolet B (UVB) radiation directly damages deoxyribonucleic acid (DNA), by crosslinking adjacent bases, forming cyclobutane pyrimidine dimers and altering the p53 tumour suppressor gene.^1,13^ The immune system plays a critical role in identifying and eliminating cancer cells, but UVB radiation can impair this response through photoimmunosuppression.^13^ Currently, ultraviolet A (UVA) is not considered a significant contributor to the development of OSSN.^1,13^ Individuals who spend 50% of their time outdoors during the first 6 years of life and those living within 30° of the equator are at greater risk of developing OSSN.^2^

Ultraviolet radiation can also reactivate HPV infections.^1,13^ Human papillomavirus is a small DNA virus with widespread asymptomatic infections. It is epitheliotropic, primarily affecting squamous epithelia, and plays a significant role in the aetiology of various squamous cell carcinomas.^13^ Human papillomavirus-induced carcinogenesis is driven mainly by the E6 and E7 proteins, which interfere with cell cycle regulation and suppress cellular repair function.^1,13^

Human papillomavirus has both mucosal and cutaneous variants and studies have yielded inconclusive findings regarding the relative strength of association between these variants and OSSN.^6,7,10,11^ The prevalence of HPV in OSSN varies significantly, with a global average of 33.8% reported previously.^7^ A recent study at Tygerberg Hospital found that 66.7% of OSSN samples tested positive for HPV, with subtypes 11, 16 and 18 being the most common.^4^ Understanding HPV types is critical when assessing public health immunisation strategies and their role in cancer prevention. Current HPV vaccines primarily target mucosal strains, and in South Africa, the bivalent vaccine only covers HPV 16 and 18. A broader vaccine effective against multiple strains could offer better protection, potentially reducing the burden of both cervical cancer and other HPV-associated cancers, such as OSSN.^4^

Immunosuppressive states, such as those seen in HIV-infected individuals and transplant patients, disrupt cancer surveillance mechanisms.^1,13^ Human immunodeficiency virus not only enhances the activity of other oncogenic viruses, including HPV but also creates a state of chronic inflammation.^1,13^ Even in patients receiving ART with undetectable viral loads, C-reactive protein (CRP) and interleukin-6 (IL-6) levels remain elevated, contributing to an inflammatory environment promoting oncogenesis.^13^

Understanding these factors is crucial for effective prevention and management strategies, particularly in high-risk populations. Continued research into the genetic and viral mechanisms behind OSSN, as well as the role of immunisation and ART, is essential to further reduce the burden of this preventable malignancy.

## Clinical presentation and diagnosis

Because of overlapping clinical features, making a clinical diagnosis of OSSN and distinguishing it from other conjunctival lesions, including pterygium, pinguecula and conjunctival papillomata, can be challenging.^6,14^ Patients typically present with similar histories of noticing a lesion, pain, foreign body sensation and redness.^2^ Benign and malignant lesions, such as pterygia and OSSN, can occasionally coexist within the same lesion. Coexisting rates exceeding 20% have been reported in South Africa and Malawi.^15^ Relying solely on clinical diagnosis can lead to inaccuracies, particularly when differentiating benign lesions from early OSSN. Therefore, histopathology is the gold standard for diagnosis.^3,11^

**FIGURE 1:**
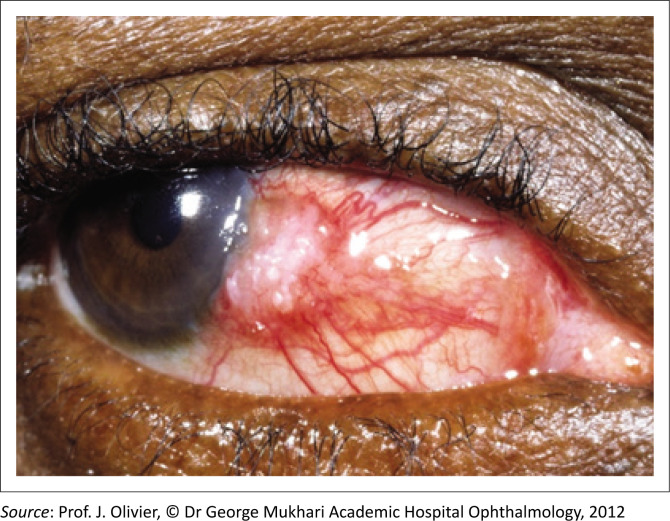
Ocular surface squamous neoplasia with raised irregular gelatinous mass, feeder vessels and leukoplakia.

**FIGURE 2:**
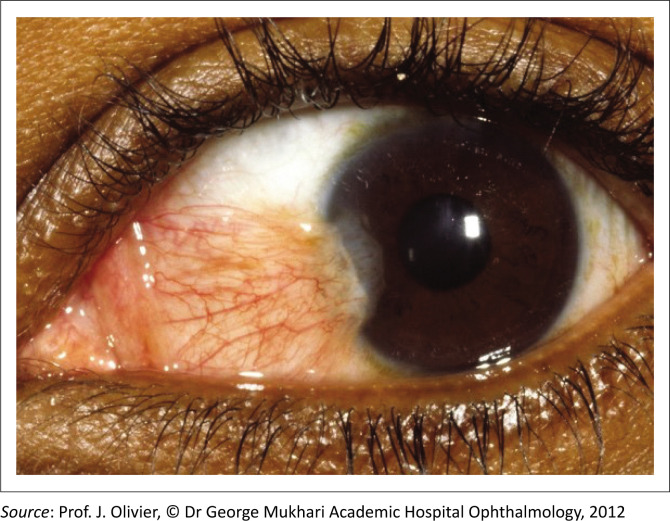
Pterygium with wing-shaped fibrovascular growth extending over cornea and absence of feeder vessels.

Clinical features that differentiate OSSN from other benign conjunctival lesions include an elevated, irregular, gelatinous or leukoplakic appearance and the presence of feeder vessels. The lesion may adhere to the sclera, show pigmentation, have a circumlimbal growth pattern and tend to be larger.^2,3^ Ocular surface squamous neoplasia is commonly unilateral,^2,10^ whereas certain benign conditions such as pinguecula are often bilateral.^14^ Figure 1 depicts an OSSN with a raised gelatinous appearance, prominent feeder vessels and leukoplakia. In contrast, Figure 2 shows a pterygium characterised by a wing-shaped fibrovascular growth extending onto the cornea. Ocular surface squamous neoplasia lesions typically occur around the nasal limbus in the interpalpebral fissure, where sun exposure is most intense,^13^ but they can extend into the fornix, especially in HIV-associated cases.^2^ Clinical characteristics that can help distinguish OSSN from other benign and malignant conjunctival lesions are summarised in Table 1.

**TABLE 1:** Clinical features, history and associated factors that can help differentiate ocular surface squamous neoplasia from other benign and malignant conjunctival lesions.

Condition	Clinical features	History and associated factors
OSSN	Lesions are elevated, irregular, gelatinous or leukoplakic Presence of feeder vessels Lesion adherent to sclera Lesion can be pigmented and larger Unilateral^2,3^	Associated with HIV, sun exposure and HPV infection^16^
Pterygium	Wing-shaped fibrovascular growth Originating from the conjunctiva or limbus, extending onto the cornea^17^	History of sun exposure^17^
Pinguecula	White-yellow elevated lesion on the nasal side of the conjunctiva Does not extend onto the cornea or cause astigmatism^18^	Associated with sun exposure, dust, wind and contact lens wear Increased incidence with age^18^
Conjunctival pyogenic granuloma	Red, smooth and lobulated vascular lesion Rapid exophytic growth Friable and may bleed easily^19,20^	Recent history of surgery or trauma More common in children and young adults^19,20^
Conjunctival nevus	Variably pigmented lesion that is flat or slightly raised Borders are well demarcated Commonly unilateral Lesion is mobile^21^	Morphological changes can be observed during puberty and pregnancy More common in Caucasians^21^
Conjunctival papilloma	Edge of lesion can be lifted (whereas OSSN tends to be part of conjunctival epithelium) Attached to a pedicle Finger-like epithelial projections surrounding vascular connective tissue core^22^	Slow growing Associated with HPV infection^22^
Kaposi’s sarcoma	Red, pink or violaceous lesion Rarely involves cornea Most commonly in the inferior fornix or bulbar area of the conjunctiva^23^	Commonly associated with HIV Associated with human herpesvirus 8 Incidence decreased with the advent of ART^23^
Conjunctival lymphoma	Pink, salmon-coloured subconjunctival mass Smooth and multilobulated More common in the fornix or mid bulbar region, less common at the limbus^24^	Affects older patients (between 60 and 70 years) Predisposing factors: Immune deficiency, autoimmune conditions, genetic mutations and immune regulation medication^24^
Conjunctival melanoma	Pigmented or non-pigmented Can contain feeder vessels and intrinsic vessels Tumour haemorrhage can occur^24^	Predisposing factors: Conjunctival nevus and primary acquired melanosis^24^

Note: Please see the full reference list of the article, Dekker L, Olivier JF, Von Pressentin K. The critical role of primary care clinicians in the early detection of ocular surface squamous neoplasia. S Afr Fam Pract. 2025;67(1), a6065. https://doi.org/10.4102/safp.v67i1.6065, for more information.

OSSN, ocular surface squamous neoplasia; HIV, human immunodeficiency virus; HPV, human papillomavirus; ART, antiretroviral therapy.

The primary care clinician’s role includes recognising the increasing incidence of these tumours, differentiating them from benign conjunctival lesions and making timely referrals. Clinicians should maintain a high index of suspicion, especially in HIV-positive patients and those with excessive sun exposure, as they are at higher risk for OSSN. Additionally, all patients presenting with OSSN should be tested for HIV, particularly those under 60 years, those with atypical or large conjunctival lesions, bilateral or multifocal tumours or a history of aggressive tumour growth.^10,25^ The high prevalence of advanced HIV disease among OSSN patients, presents an opportunity to identify individuals who would benefit from life-saving ART, especially those without other clinical manifestations of HIV.^8^

A thorough medical history should include inquiries about multiple sexual partners and a history of sexually transmitted diseases, particularly HPV infections.^26^ During a general examination, clinicians should assess for UV-related malignancies, such as squamous and basal cell carcinoma of the skin,^27^ as well as other signs of HIV. Although lymphadenopathy is rare, even in advanced OSSN,^3^ submandibular and submental lymph nodes should still be examined. A comprehensive eye examination should include an assessment of both eyes, with detailed documentation of the lesion and its extent. Visual acuity should be tested with and without a pinhole to evaluate for astigmatism, and fluorescein staining should be used to assess corneal and conjunctival epithelial staining defects (see Figure 1 and 2).

Access to specialised ophthalmic services and equipment, such as a slit-lamp, to facilitate an appropriate diagnosis may be restricted, particularly in low- and middle-income countries. However, with the current cohort of optometry graduates having diagnostics skills, especially in South Africa, it may be advisable to consider their services as they may be readily available in some instances. Point-of-care technology may assist primary care clinicians in the early detection of malignant eye conditions, facilitate a correct diagnosis and appropriately refer patients to eye care practitioners, especially ophthalmologists. Artificial intelligence (AI) based diagnostic systems such as smartphone cameras may enhance the screening process and complement the clinical assessment performed by primary care clinicians. While numerous machine learning studies have concentrated on skin melanoma, training conventional deep learning networks to detect lesions such as conjunctival melanoma and OSSN poses challenges because of the lack of extensive datasets containing images of conjunctival disorders.^28^ However, as AI-associated technologies advance, the early detection of OSSN may be facilitated, leading to better treatment outcomes. Artificial intelligence algorithms can use a combined appraisal of patient data, including genetics, medical history and lifestyle factors, to identify individuals at a higher risk of developing OSSN.^26^

Furthermore, teleophthalmology is emerging as a vital tool in underserved communities worldwide. In South Africa, the Vula mobile app connects rural patients with specialist care and provides virtual outreach in resource-constrained settings. Managing patients at local healthcare facilities benefits both patients and the healthcare system. Timely and appropriate management also significantly improves patient outcomes.^27^ A study in rural Australia showed that optometrist-led teleophthalmology boosted surgical case rates and reduced wait times, demonstrating significant economic benefits and enhanced care coordination.^29^

## Treatment modalities

Although ophthalmologists typically manage treatment, primary care clinicians must better understand the available options to educate and support patients. Treatment primarily consists of surgical excision, topical chemotherapy and occasionally radiotherapy.^11^ When the extent of local spread is uncertain, imaging modalities such as computed tomography scans can be used to evaluate it further.^10^

Surgical excision may be combined with intraoperative cryotherapy, which can disrupt tumour cell membranes and block feeder vessels, reducing the risk of recurrence.^2^ There is an increased need for aggressive surgical interventions, such as enucleation and exenteration, in HIV-associated tumours because of their invasiveness.^10,25^

Also, there is an increasing trend towards medical management to avoid surgical complications. To this end, topical agents such as 5-fluorouracil (5-FU), mitomycin C (MMC) and interferon alfa (IFNα) are being used as monotherapy. These agents effectively treat visible lesions and subclinical disease across the ocular surface.^11^

## Conclusion

Africa has high rates of HIV, HPV infection and year-round sun exposure, all contributing to the rising incidence of OSSN.^6,8^ Patients with HIV often present at a younger age with more aggressive forms of the disease. Given the high prevalence of advanced HIV among those with OSSN, it is crucial to screen all OSSN patients for HIV. This screening not only facilitates early diagnosis but also provides an opportunity to initiate life-saving ART in asymptomatic individuals. Despite the inconclusive impact of ART on OSSN occurrence, the chronic management of HIV is likely to increase the number of OSSN cases encountered in clinical practice.

It is therefore critical for primary care clinicians to understand the risk factors for OSSN, identify at-risk patients and recognise its clinical features to differentiate it from benign conjunctival lesions, such as pterygia. Vigilance in early detection and prompt referral are essential to prevent complications such as local infiltration, blindness and exenteration.
